# The effect of cheats on siderophore diversity in *Pseudomonas aeruginosa*

**DOI:** 10.1111/jeb.13307

**Published:** 2018-07-02

**Authors:** Peter Stilwell, Chris Lowe, Angus Buckling

**Affiliations:** ^1^ Biosciences University of Exeter Cornwall UK

**Keywords:** cheating, cooperation, Greenbeard effect, public goods

## Abstract

Cooperation can be maintained if cooperative behaviours are preferentially directed towards other cooperative individuals. Tag‐based cooperation (greenbeards) – where cooperation benefits individuals with the same tag as the actor – is one way to achieve this. Tag‐based cooperation can be exploited by individuals who maintain the specific tag but do not cooperate, and selection to escape this exploitation can result in the evolution of tag diversity. We tested key predictions crucial for the evolution of cheat‐mediated tag diversity using the production of iron‐scavenging pyoverdine by the opportunistic pathogen, *Pseduomonas aeruginosa* as a model system. Using two strains that produce different pyoverdine types and their respective cheats, we show that cheats outcompete their homologous pyoverdine producer, but are outcompeted by the heterologous producer in well‐mixed environments. As a consequence, co‐inoculating two types of pyoverdine producer and one type of pyoverdine cheat resulted in the pyoverdine type whose cheat was not present having a large fitness advantage. Theory suggests that in such interactions, cheats can maintain tag diversity in spatially structured environments, but that tag‐based cooperation will be lost in well‐mixed populations, regardless of tag diversity. We saw that when all pyoverdine producers and cheats were co‐inoculated in well‐mixed environments, both types of pyoverdine producers were outcompeted, whereas spatial structure (agar plates and compost microcosms), rather than maintaining diversity, resulted in the domination of one pyoverdine producer. These results suggest cheats may play a more limited role in the evolution of pyoverdine diversity than predicted.

## Introduction

Cooperation, a behaviour that has at least in part evolved because it benefits others, can be evolutionarily unstable because noncooperative organisms can exploit cooperators. Cooperation can, however, be maintained if the benefits of cooperation can be directed towards other cooperators. This typically occurs when cooperative interactions occur more frequently between genealogical kin, as a result of either population structure or kin recognition (Hamilton, 1964; Lehmann & Keller, 2006; West *et al*., 2007), but can also arise if the cooperative trait is encoded together with a trait confining the benefits of cooperation to individuals carrying the same cooperation genes (‘greenbeard genes’) (Dawkins, 1976; West & Gardner, 2010; Biernaskie *et al*., 2011, 2013). Although originally thought to be very rare, recent years have uncovered more and more examples of both helping and harming greenbeard genes (the latter encoding harming behaviour towards individuals without the greenbeard (Gardner & West, 2010; Biernaskie *et al*., 2013), in organisms including lizards, fire ants, bacteria, yeast and amoeba (Keller & Ross, 1998; Queller *et al*., 2003; Sinervo *et al*., 2006; Smukalla *et al*., 2008; Inglis *et al*., 2009).

There is also growing evidence that in some organisms, multiple ‘beard colours’, or tags, exist (Smukalla *et al*., 2008; Bodilis *et al*., 2009). Such diversity can theoretically be explained by the evolution of ‘falsebeards’ – cheats that are able to receive the benefits of directed cooperation without paying the cost of the cooperative behaviour and thus outcompete their cooperative greenbeard counterparts (Jansen & van Baalen, 2006; Rousset & Roze, 2007; Lee *et al*., 2012). This effect relies on interactions occurring within patches in structured populations. Within a given patch, a cooperative tag type that is not exploited will outcompete exploited tag types and their associated cheats. Stochasticity associated with structured populations can result in the co‐occurrence of tag types (and their cheats) to vary in space and time, so different tag types will win in different patches. Crucially, when a tag type is rare in the structured populations, its associated cheats will also on average be rare, resulting in a fitness advantage to rare cooperator tag types which can then result in stable coexistence of multiple tag types (Lee *et al*., 2012). Note that in the absence of population structure, such that multiple tag types and their respective cheats are present in all patches, the maintenance of tag‐based cooperation is unlikely (Rousset & Roze, 2007). This is because the advantage of being a rare tag type with few cheats in a patch is offset by the advantage of being a common tag type that necessarily receives more helping behaviour, eliminating the negative frequency dependence required for the maintenance of diversity.

Here, we experimentally determine whether the presence of cheats can explain the maintenance of tag diversity in a potential multitag system: production of pyoverdine, a siderophore, by the bacterium *Pseudomonas aeruginosa*. Iron is essential for almost all forms of life yet is limited in many environments due to its poor solubility in ferric form (Andrews *et al*., 2003). Siderophores are compounds secreted by bacteria to scavenge insoluble or host‐bound iron (Cornelis, 2010). Pyoverdine, a fluorescent peptide with high affinity for iron, is the main siderophore produced by *P. aeruginosa* (Meyer *et al*., 1996). Pyoverdine is costly to make (Griffin *et al*., 2004), and pyoverdine‐iron complexes can be used by any cells with an appropriate receptor (Hohnadel & Meyer, 1988; Cornelis *et al*., 1989; Smith *et al*., 2005), hence pyoverdine can be a cooperative trait and producers can be invaded by cheats (West & Buckling, 2003; Griffin *et al*., 2004; Harrison *et al*., 2006; Buckling *et al*., 2007; Harrison & Buckling, 2009). Broadly, three types of pyoverdine and receptor pairs have been described to date (Cornelis *et al*., 1989; Meyer *et al*., 1997; De Vos *et al*., 2001; Bodilis *et al*., 2009), and cross‐feeding, binding and uptake assays suggest that pyoverdine‐mediated iron transport is relatively specific to the pyoverdine‐receptor combination (Hohnadel & Meyer, 1988; Cornelis *et al*., 1989). Single strains seem to produce only one pyoverdine type, although a degree of generalism can be achieved by production of multiple receptors (Ghysels *et al*., 2004). The dynamics of pairwise interactions between co‐existing *Pseudomonas* species, encoding different pyoverdine types, and at different levels of expression, have been recently explored. Recent work suggests that nonproduction of pyoverdine may be a driver of pyoverdine diversity, although the mechanism remains undetermined (Butait≐ *et al*., 2017).

We first determine whether the within‐patch competitive outcomes required for cheat‐mediated maintenance of tag diversity hold for pyoverdine diversity, namely (1) tag‐based cooperators are outcompeted by their tag‐specific cheats, but outcompete cheats associated with a different tag; and (2) a specific cooperator tag type without cheats will outcompete other cooperator tag types and their associated cheats. We then test the predictions that (3) tag‐based cooperation will be selected against when each cooperator tag type has its cheat present in well‐mixed environments, whereas (4) diversity of tag‐based cooperation will be maintained if all cheat types are present in spatially structured environments. To this end, we conduct short‐term experiments *in vitro* as well as longer term experiments in soil‐based growing media (compost) (Gómez & Buckling, 2013).

## Materials and methods

### Bacterial strains

*Pseudomonas aeruginosa* strains PA01 and 59.20 produce siderophore types I and III, respectively, each expressing a surface receptor cognate with their siderophore type (*fpvAI* in the case of PA01 and *fpvAIII* in the case of 59.20) (De Chial *et al*., 2003). Cultures of PA01 and 59.20 were transferred and plated every 2 days in iron‐limited casamino acid media (CAA) (see below) until a non‐siderophore‐producing mutant evolved. Experiments were conducted in iron‐limited media to stimulate siderophore production and ensure that the trait is a cooperative behaviour. After 10–20 2‐day transfers (depending on strain), one non‐siderophore‐producing mutant colony from each strain was picked based on loss of yellow‐green pigmentation. As pyoverdine is a green, fluorescent siderophore, nonproducing mutants (cheats) are demarcated from pyoverdine‐producing strains (cooperators) by their colonies’ lack of pigmentation when grown on King's B (KB) agar. Picked colonies were then grown in CAA, and each 1 mL was mixed with glycerol and frozen at −80 °C. Reduced *per capita* iron chelator activity of these mutants compared to the cooperators from which they were derived was confirmed with the chrome azurol S (CAS) assay, as described in Harrison & Buckling (2007). Both PA01 and 59.20 cooperator strains were found to have significantly higher per capita iron chelator activity (PA01; *t*_10_ = 14.038, *P* < 0.0001; 59.20; *t*_10_ = 10.664, *P* < 0.0001) (see Figure S1).

To unambiguously distinguish strains in pairwise competitions, both the PA01 cheat and 59.20 cooperator were transformed with the *lacZ* operon (following (Choi *et al*., 2006)). Briefly, to render bacteria electro‐competent, populations were grown overnight in 6 mL Luria–Bertani (LB) medium, harvested by centrifugation (2 min at 18,000 *g*), washed twice with 1 mL 300 mm sucrose and then finally resuspended in 100 *μ*L room temperature 300 mm sucrose. For transformation, 500 ng of purified plasmid DNA was mixed with 100 *μ*L electrocompetant cells and transferred to a 2 mm gap width electroporation cuvette (Bio‐Rad). A pulse of 2.5 kV was applied to the cells, after which 1 mL of LB was used to wash the cells from the cuvette. This wash was then incubated for 1 h at 37 °C, shaken at 180 r.p.m. One hundred microlitres of this final culture was plated onto an LB + Gm (30 *μ*g mL^−1^) + X‐gal (20 *μ*g mL^−1^) plate. Colonies with the LacZ phenotype were blue on X‐gal (20 *μ*g mL^−1^ final concentration) plates. Note that for the colony selected for the experiment, there was no detectable growth rate cost associated with insertion of the *lacZ* operon under the culture conditions described below (59.20, with and without *LacZ*,* T*_10_ = 1.314 *P* = 0.218; PA01, with and without *LacZ*,* T*_10_ = 1.153, *P* = 0.276, see Figure S2).

We used colony PCR in conjunction with the LacZ phenotype to distinguish strains in three‐ and four‐way competition experiments. Populations were plated onto KB agar containing 30 *μ*g mL^−1^ X‐gal and incubated overnight at 37 °C. Colonies from these plates were picked into 75 *μ*L Milli‐Q H_2_0. One microlitre of this was used as template for a PCR: in a total volume of 10 *μ*L, 5 *μ*L of DreamTaq Green PCR Master Mix (ThermoFisher) (Thermo Fisher Scientific, MA, USA), with 0.25 *μ* m of each forward and reverse primers for PA01 and 59.20 ferripyoverdine genes *FpvAI* and *FpvAIII* (De Chial *et al*., 2003), respectively, the remaining volume made up with H_2_O. The thermocycler was run with the following parameters: 96 °C for 10 min, followed by 35 cycles of 95 °C for 30 s, 55 °C for 30 s, and 72 °C for 30 s, with a final extension of 7 min at 72 °C. Two distinct bands, of ~ 300 and ~ 500 bp, corresponding to *fpvA* types I and III from PA01 and 58.20, respectively, were observable following electrophoresis (1.2% agarose in TAE, 35 min at 120 V).

### Culture conditions

Cultures were streaked on KB agar, and six colonies from each strain were used to establish six replicates for each competition experiment. These starting cultures were grown overnight at 37 °C in 30‐mL glass universal tubes containing 6 mL (KB) on a 180 r.p.m. orbital shaker. One millilitre of each culture was pelleted by centrifugation at 15,000 *g* for 5 min at room temperature, and the supernatant was removed and the pellet resuspended in 1 mL of M9 salts (70 g Na_2_HPO_4_•7H_2_O, 30 g KH_2_PO_4_, 5 g NaCl, 10 g NH_4_Cl). Suspensions were diluted in M9 salts to OD 600 nm 0.2 (~ 10^8 cfu mL^−1^). Strains were then combined as appropriate for the specific experiment and treatment, described below.

In broth experiments (1–4, below), 60 *μ*L from each mix was inoculated into 6 mL of casamino acids (CAA) media (5 g casamino acids, 1.18 g K_2_HPO_4_•3H_2_O, 0.25 g MgSO_4_•7H_2_O, 1 L H_2_O), supplemented with 100 *μ*g mL^−1^ human apo‐transferrin (Sigma, Gillingham, UK), an iron chelator, and 20 mm NaHCO_3_ (sodium bicarbonate), required for iron chelator activity (Meyer *et al*., 1996). Cultures were plated immediately after inoculation and incubated at 37 °C in static, 30‐mL glass universal tubes. Every 24 h, cultures were plated and 1% transferred into new iron‐limited CAA media, for six transfers, or until a strain was no longer detectable.

#### Monoculture growth

Each strain was grown as a monoculture, and Malthusian parameters ((*m *= ln(final density/starting density) (Lenski *et al*., 1991)) of cooperators and corresponding cheats compared using *t*‐tests.

#### Pairwise competitions

To investigate the performance of cheats in the background of either the strain from which they were derived (their homologous strain) or the alternative siderophore‐type producer (their heterologous strain), four‐two‐way competition treatments were set up competing each cooperator strain against each cheat at equal initial frequencies. The relative fitness (*w*) of each cheat strain in each pairwise competition was calculated from the ratio of each strain's 24‐h Malthusian growth parameter (*m*), averaged through time (*m* was calculated for each strain within a replicate at each 24‐h time point). Fitness differences between competitors were determined by carrying out one‐sample *t*‐tests.

#### Three‐way competitions

To determine whether being cheat‐free allows one cooperator strain to outcompete both the other cooperator strain and its associated cheat, we competed both cooperators with one or other of the cheats. Strains were inoculated at roughly equal initial frequencies, and the relative fitness (*w*) of the nonexploited cooperator was determined with respect to growth of the total competitor population and analysed as above.

#### Effect of cheats on the maintenance of diversity in well‐mixed environments

To test whether the presence of cheats affected the diversity of strain types when competing directly in well‐mixed environments, after mixing at roughly equal initial frequencies, we determined the frequency of each type every 24 for 144 h when all four strains were competing and when only the two producing strains were competing. This experiment was replicated in two blocks because of high within‐treatment variation. We estimated diversity as the proportion of the rarest strain background (i.e. summing together strain, regardless of social strategy; PAO1 and its cheats, and 59:20 and its cheats in the four‐way competition experiments). Proportion of rarest strain was used as the response variable in a linear mixed model, with treatment (with or without cheats present), time, experimental block and the treatment by time interaction fitted as explanatory variables, and replicate as a random factor nested within treatment. Note that we sum cheats and cooperators because diversity of cooperator types is also associated with diversity of cheat types (Lee *et al*., 2012). However, we obtain the same qualitative conclusions throughout when we use cooperator diversity only as a metric. Other measures of diversity (the Simpson (Simpson, 1949) and Shannon (Shannon, 1948) diversity indices) also lead to the same conclusions.

#### Effect of cheats on the maintenance of diversity on agar plates

To test whether cheats could maintain cooperator diversity in spatially structured environments, we conducted invasion‐from‐rare competition experiments on agar plates. Soft agar provides structure to the environment, which precludes the formation of colonies, and limits the diffusion of siderophore (Kümmerli *et al*., 2009). Briefly, to 1 L of CAA, either 12 g (hard agar) or 6 g (soft agar) of agar was added and autoclaved. After cooling to 45 °C, media were supplemented with 100 *μ*g mL^−1^ apo‐transferrin and sodium bicarbonate at 20 mm (as per (Kümmerli *et al*., 2009)). On the day of the experiment, into 10 cm petri dishes ~ 20 mL of hard agar was poured and allowed to set. To 2.5 mL of soft agar, 500 *μ*L of competition populations was added, containing a total of 10^3^ c.f.u. of the following strain mixes: PA01 cooperator at approximately 1% with 59.20 cooperator at 99%; PA01 cooperator and cheat each at 0.5%, with 59.20 cooperator and cheat each at 49.5%; 59.20 cooperator at 1% with PA01 cooperator at 99%; and 59.20 cooperator and cheat each at 0.5% with PA01 cooperator and cheat at roughly 49.5%. The soft agar + inoculum mix was briefly pulsed on a vortex before being poured onto a hard agar dish, and then allowed to set before being sealed with parafilm, inverted and incubated for 6 days at 37 °C. After 6 days, the soft agar surface was repeatedly rinsed five times with a 10 mL volume of M9 using a serological pipette and pipette controller. Inocula and rinses were plated, and colonies counted according to their LacZ phenotype. The relative fitness values of rare cooperator strains were assessed using one‐sample *t*‐tests.

#### Effect of cheats on the maintenance of diversity in structured and unstructured compost microcosms

To determine the importance of cheats and spatial structure in maintaining cooperator diversity under more natural conditions, we competed strains in static compost‐water and shaken compost‐water microcosms (Gómez *et al*., 2015; Lujan *et al*., 2015). Six grams of compost (John Innes no. 2) was aliquoted in 30‐mL glass universal tubes and autoclaved. To avoid potential contamination from surviving fungal spores, these microcosms were autoclaved a second time after 2 days at room temperature. Five millilitres of autoclaved Milli‐Q water was added to these tubes prior to inoculation. Six replicate microcosms were inoculated in four treatment groups; both cooperator types alone at 50% each, and both cooperators with their homologous cheats at roughly 25% each, in either static or shaken (180 r.p.m.) conditions. Tubes were vortexed at maximum speed for 15 s, and 60 *μ*L was transferred to fresh soil microcosms each week for 6 weeks. Diversity of strain types (regardless of social strategy) was estimated by scoring colonies for their *LacZ* phenotype from populations plated after inoculation, after growth in the third transfer, and after growth in the final transfer. Strain proportions were fitted by REML, with the factors time, population structure (static or shaken microcosms) and the presence or absence of cheats, with replicate modelled as a random effect. The CAS assay was used to determine per capita iron chelator activity at the end of the experiment (because evolutionary change in siderophore production arsing by *de novo* mutation may have occurred over the time scale of the experiment): 20 *μ*L of each replicate culture was inoculated into two wells of a 96‐well plate containing 180 *μ*L of iron‐limited CAA (CAA supplemented with 100 *μ*g mL^−1^ human apo‐transferrin and 20 mm NaHCO_3_, which is required for iron chelator activity (both from Sigma)). Iron chelator activities were compared using a GLM, with the factors strain (PA01 and 59.20), population structure (shaken and static) and starting social strategy (cooperator or cheat).

## Results

### Growth cost of cheats in monoculture

Both cooperators had a greater growth rate than their respective cheats (PAO1: *t*_10_ *= *7.089, *P* < 0.0001; 59.20: *t*_10_ *= *2.457, *P* = 0.0378) in monoculture, demonstrating a growth rate cost of the non‐pyoverdine‐producing mutants under iron‐limited conditions.

### Cheat specificity

**Figure 1:**
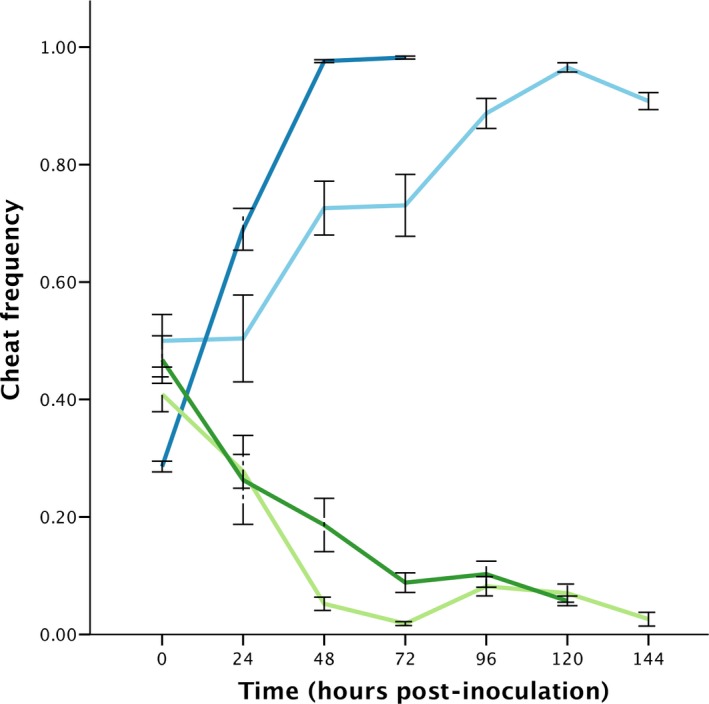
Cheat frequency over time. Cheats dominate the population in the presence of the homologous cooperator from which they were derived (blues) and are eliminated from the population when in competition with a heterologous cooperator (greens). Light blue and light green show 59.20 cheat in the presence of 59.20 cooperator and PA01 cooperator, respectively. Dark blue and dark green show PA01 cheat in the presence of PA01 cooperator and 59.20 cooperator, respectively. Error bars show mean ± SEM.

In two‐way competition, the PA01 cheat had a higher fitness than PAO1 cooperator (Fig. 1; *t*_5_ = 74.538, *P* < 0.001) and a lower fitness than the 59.20 cooperator (*t*_5_ = −12.86, *P* < 0.001). Similarly, the 59.20 cheat had a higher fitness than 59.20 cooperator (*t*_5_ = 9.676, *P* < 0.001) and a lower fitness than PAO1 cooperator (*t*_5_ = −8.419, *P* < 0.001). At the final time point, cooperators in all six replicate populations from both homologous competitions had been eliminated, whereas in heterologous competitions, cheats had been eliminated from 6/6 replicates in the case of PA01 cheat vs. 59.20 cooperator, and 5/6 in the case of 59.20 cheat vs. PA01 cooperator. These results indicate that cheats were only cheats with respect to their homologous cooperator.

### Cooperators with no cheats outcompeted cooperator–cheat combinations

**Figure 2:**
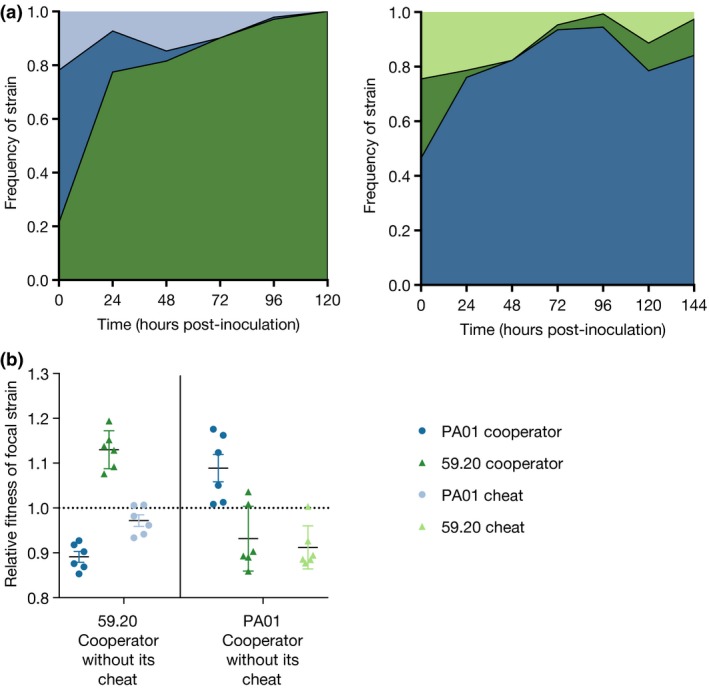
(a) Average frequency of strains in three‐way competitions. The outcome of competition between two cooperating strains is strongly influenced by the type of cheat also present in the population. The frequency of PA01 cooperator is shown in dark blue, 59.20 cooperator in dark green, with PA01 and 59.20 cheats in light blue and light green. Error bars show ± SD. (b) Relative fitness (Malthusian parameter of focal species relative to the Malthusian parameter of the combination of the other competitors) averaged over time of all strains in the two‐three‐way competitions. Error bars show mean ± SEM.

The PAO1 cooperator outcompeted the combination of cooperator and cheat 59.20 (Fig. 2; *t*‐test, *t*_5_ = 2.919, *P* = 0.033), and went to fixation in 6/6 replicates. The 59.20 cooperator outcompeted the combination of PAO1 cooperator and its cheat (Fig. 2; *t*‐test, *t*_5_ = 7.50, *P* < 0.001), going to fixation in 6/6 replicates. These results show that the absence of a cheat allows a cooperator strain to outcompete other cooperator–cheat combinations.

### Cheats did not maintain diversity of siderophore types in broth

**Figure 3:**
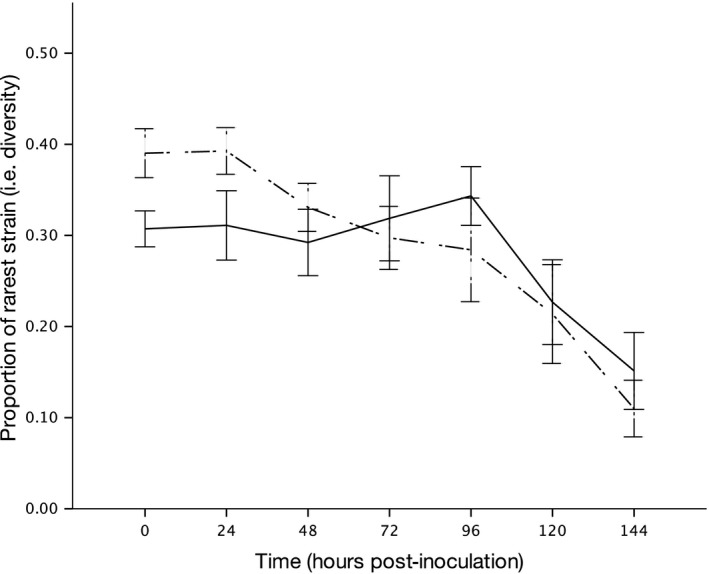
Mean proportion of rarest strain. Cooperators and cheats of the same strain type in the ‘With Cheat’ treatments were summed, and the proportions of each strain were calculated for both treatments. The proportion of the rarest strain in each replicate was taken as a crude measure of diversity. Dashed line represents populations without cheats, and solid line represents populations with cheats. Error bars show mean ± SEM.

Here, we looked at the effect of both cheats on diversity in populations of the two cooperators by comparing the diversity of strains through time in treatments with and without cheats, in the absence of spatial structure. The presence of cheats had no effect on the mean proportion of the rarest siderophore‐type producer in a population (Fig. 3; treatment: *F*_1,21_ = 0.4582, *P* = 0.506). Diversity decreased through time in both treatments (time: *F*_1,142_ = 59.576, *P* < 0.001), but we did not observe competitive exclusion in most cases. The presence of cheats did not have a different effect in the two experimental blocks (block by treatment interaction: *F*_1,20_ = 0.425, *P* = 0.522).

### Cheats did not maintain diversity on agar plates

**Figure 4:**
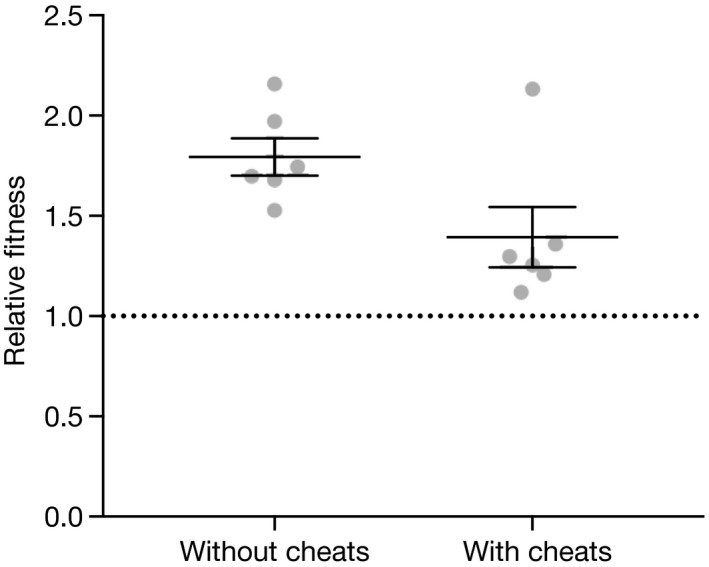
Relative fitness of rare PA01 in soft agar overlays. 59.20 was undetectable in both treatments. The horizontal line at 1 indicates equal fitness of the rare and common strain (*W* = 1). Error bars show mean ± SEM.

Here, we looked at the effect of both cheats on diversity in populations of the two cooperators by comparing the diversity of strains through time in treatments with and without cheats, in a spatially structured environment. Strain 59.20 failed to invade from rare in the presence and absence of cheats, and at the end of the experiment was below the limit of detection in all replicates of both treatments. Strain PA01 had a higher relative fitness (compared to 59.20) when rare both in the presence and absence of cheats (one‐sample *t*‐test against a hypothesized mean of 1; cheats present, *t*_5_ = 2.616, *P* = 0.047; cheats absent *t*_5_ = 8.551, *P* = 0.0004). Together, these data indicate that diversity will always be lost in these competitions: PA01 will outcompete 59.20 regardless of whether it is rare or common, and regardless of whether cheats are present or absent. See Fig. 4.

### Cheats did not maintain diversity in unstructured or structured compost

**Figure 5:**
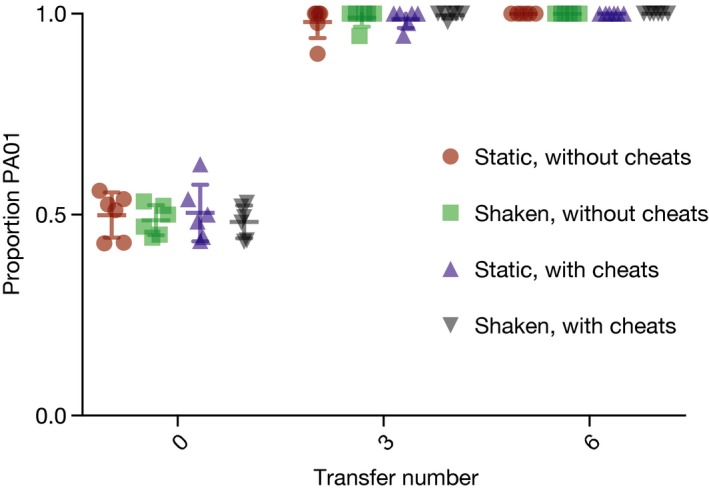
Proportion of strain PA01 in the population, with and without cheats, in shaken and static compost microcosm environments. Error bars show mean ± SEM.

In competitions in compost between the two cooperators, with and without cheats, diversity decreased through time in all populations (*F*_1,47_ = 146.393, *P* < 0.0001). The rate of diversity loss was not altered by shaking the environments (*F*_1,22_ = 0.114, *P* = 0.739), the presence of cheats (*F*_1,22_ = 0.128, *P* = 0.724), nor any effect of the presence of cheats acting differently in shaking or static environments (population structure by cheat presence interaction_,_ *F*_1,20_ = 0.068, *P* = 0.797). As with the competitions carried out on plates, strain PA01 was dominant in all treatments by the final time point. See Fig. 5.

### Cooperation was maintained in structured, but not unstructured compost

**Figure 6:**
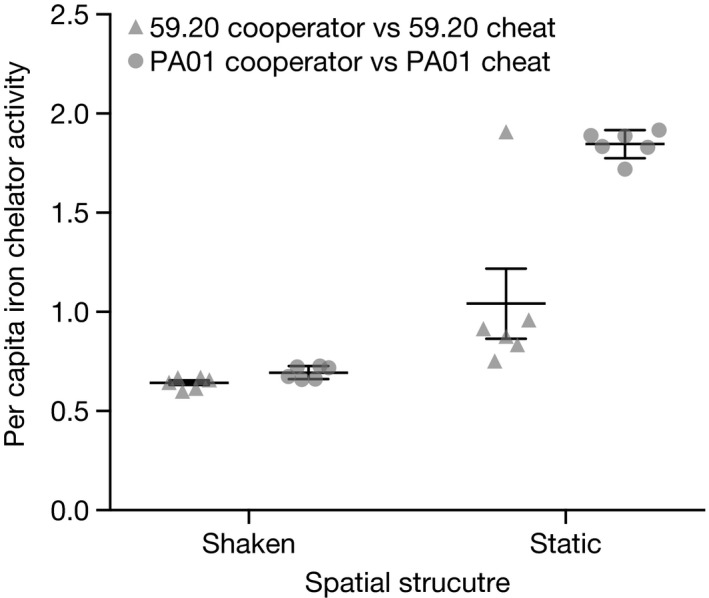
Per capita iron chelator activity of populations from competitions between cooperators and their respective cheats in shaken and static compost microcosms, taken at the final time point. Data points show the average of two technical replicates. Error bars show mean ± SEM.

In competitions between cooperators and their respective cheats, per capita iron chelator activity was higher in static environments than shaken (*F*_1,20_ = 75.089, *P* < 0.0001). There was no difference in final per capita iron chelator activity between strains in the shaken compost (*F*_1,20_ = 0.166, *P* = 0.689), whereas iron chelator activity was higher in PA01 than 59.20 in static compost (*F*_1,20_ = 40.413, *P* < 0.0001). See Fig. 6.

## Discussion

We determined from competition experiments between two types of pyoverdine producer and their respective cheats whether cheats can potentially maintain the diversity of *P. aeruginosa* pyoverdine ‘greenbeard’ alleles. Cheats were able to outcompete their homologous cooperators, but cooperators were able to outcompete the heterologous cheats. Moreover, when multiple tag types and cheats were present in a patch, tags without cheats outcompete other strains. These data are consistent with theory that suggests cheating can play a role in maintaining tag diversity and cooperation in spatially structured environments (Smith *et al*., 2005; Rousset & Roze, 2007; Lee *et al*., 2012), where not all combination of tag types and cooperator/cheat strategies are present in every patch. Specifically, the pairwise competitions within patches resolve in exactly the manner assumed in theory. However, when both cheat and both cooperator types were present, we did not find evidence that tag diversity could be maintained by such a mechanism: Cheats were not able to maintain diversity in two spatially structured environments: agar plates and compost microcosms, where instead one of the strain types (PAO1) consistently dominated.

These results may suggest that cheats play a more limited role in the evolution of tag diversity than expected from recent theory: Although each of the two and three‐way competitions in broth showed the predicted effect, the superior competitive ability of PA01 was not overcome in the presence of both its cheat together with the alternative cooperator and cheat. Many inequalities between the strains – which is likely to be the case in nature – could affect the robustness of theoretical predictions. For example, fitness differences between the two cooperator types and two cheat types due to intrinsic growth rate differences, siderophore cost differences or variation in siderophore‐iron affinity, may all reveal a limited parameter space in which this effect can be found. One further consideration absent from theoretical predictions is the ability of siderophores to act as competitive traits (Niehus *et al*., 2017). Significant investment in production of siderophore in lieu of growth may, in environments of finite iron availability, limit the ability of cheats to establish the niche an alternative competitor requires. Further experiments involving other *P. aeruginosa* strain combinations, or strains genetically modified to express different siderophore types, may show the predicted effect and reveal the extent to which social interactions play a part in the maintenance of cooperation and diversity.

Theory suggests that in the absence of sufficient population structure tag diversity will be eroded (Rousset & Roze, 2007), because the maintenance of diversity relies on different combinations of tag types and their cheats in different patches, and the subsequent migration between patches (Lee *et al*., 2012). It is therefore possible that the spatial structure we imposed did not generate such conditions. However, this seems unlikely: we saw that cheats did not dominate spatially structured compost microcosms as they did in well‐mixed broth environments.

In contrast to our work, recent research has also shown that diversity can be maintained within patches by a single cheat: a so‐called loner effect. A dynamic polymorphism of two cooperators with different tags and a single cheat was maintained due to the cheat equalizing the fitness of the two cooperators by exclusively targeting the more competitive cooperator (Inglis *et al*., 2016): Cheats outcompeted their cooperator strain; the loner strain (the less competitive cooperator) outcompeted the cheat, and the better cooperator outcompeted the loner strain. The strains used in this experiment are similar to the ones used in left panel of Fig. 2a, with a PA01 cooperator/cheat pair, and a type‐III siderophore producer; the same type produced by our 59.20 cooperator. The difference between the results probably reflects the differences in the costs of siderophore production, the proportion of siderophores shared or proportion retained for personal use (see Inglis *et al*., 2016), which in our case did not have suitable values for the loner effect to occur.

Recent work investigating the dynamics of co‐occurring natural *Pseudomonas* isolates indicate that selection for cheating, and corresponding resistance to cheating, could drive pyoverdine diversification (Butait≐ *et al*., 2017). The results from our pairwise competitions would lead us to similar conclusions. However, extending our work to multiple producer and cheat strain competitions shows that although nonproduction does exert a selective pressure, the cooperative social dynamics of pyoverdine production alone may be insufficient to maintain diversity. The existence of strains that can take up multiple pyoverdines ‘(multibeards’) also questions the role of cheats in driving pyoverdine diversity. Certain strains of *P. aeruginosa*, particularly type‐II pyoverdine producers, are able to take up type‐I pyoverdines (De Vos *et al*., 2001) through a second receptor for type‐1 pyoverdine, *Fpv‐B* (Ghysels *et al*., 2004), and receptors of type‐III pyoverdine can recognize pyoverdine type‐II (Ghysels *et al*., 2004). Why the ability to take up all of a competitor's pyoverdines is not a ubiquitous trait is unclear, but one evolutionary constraint acting on this ‘multibearded’ phenotype may be increased susceptibility to bacteriocins. Pyocin S3, a bacteriocin of *P. aeruginosa*, uses pyoverdine type‐II as its receptor (Baysse *et al*., 1999), whereas pyocin S2 kills strains via *FpvA* receptor type‐I (Denayer *et al*., 2007). This functional link between pyocins and pyoverdines in the form of the receptor may be the driver of diversity in these traits, rather than cheat interactions. Alternatively, different pyoverdine types may simply be beneficial in different ecological contexts, as have recently been shown for the different siderophore classes produced by *P. aeruginosa* (Dumas *et al*., 2013).

Diversity of helping tags is present in other microbial systems, and further work is needed to determine whether cheating plays a role in their evolutionary maintenance. For example, flocculation – a stress resistance aggregation phenotype – in *Saccharomyces cerevisiae* is encoded by the highly variable *FLO1* gene with individuals not expressing the gene excluded from the aggregate (Brown & Buckling, 2008; Smukalla *et al*., 2008). The cell–cell adhesion property encoded by *FLO1* correlates with the number of tandem repeats within the gene; it is conceivable that greenbeard cheating may manifest in the form of yeast expressing *FLO1* with a low number of tandem repeats, maintaining the benefit of inclusion in the aggregate whilst lowering the fitness cost of *FLO1* expression (Brown & Buckling, 2008). Cheating may also play a role in the maintenance of diversity of harming tags. For example, *Escherichia coli* produce a diversity of colicins, a plasmid‐encoded bacteriocin which kills nonplasmid carrying individuals (James *et al*., 1996), with positive selection acting at the colicin and immunity loci (Riley, 1993). Immune, but non‐colicin‐producing mutants can invade colicin producers, but are outcompeted by sensitive nonproducer (Kerr *et al*., 2002), suggesting that immune, colicin nonproducing, greenbeard cheats may also have imposed selection for diversity in colicins (Pagie & Hogeweg, 1999; Biernaskie *et al*., 2013).

In summary, we failed to provide a direct experimental demonstration that cheats play an important role in generating and maintaining diversity, and show the potential problems of inferring the evolution and maintenance of tag diversity from the outcome of simple competition experiments. Differences between strains over and above cooperator tag type no doubt contributed to the failure of cheats to maintain diversity, but some differences are of course the norm in natural populations. Models incorporating between‐strain inequalities in intrinsic growth, siderophore expression and siderophore‐iron affinity are needed to explore the robustness of the predicted effect of cheats.

## Supporting information


**Figure S1** Results of CAS assay showing per capita iron chelator activities of PA01 WT (cooperator), PA01 cheat, 59.20 WT (cooperator), and 59.20 cheat.
**Figure S2** Malthusian parameters of strains 59.20 and PA01 with and without the *LacZ* marker inserted.
**Table S1** Colony counts, and calculated Malthusian parameters of PA01 cooperator and cheat in competition.
**Table S2** Colony counts, and calculated Malthusian parameters of PA01 cooperator and 59.20 cheat in competition.
**Table S3** Colony counts, and calculated Malthusian parameters of 59.20 cooperator and 59.20 cheat in competition.
**Table S4** Colony counts, and calculated Malthusian parameters of 59.20 cooperator and PA01 cheat in competition.
