# Supplementary material for: The effect of cheats on siderophore diversity in Pseudomonas aeruginosa
Source: J Evol Biol. 2018 Jul 2;31(9):1330–9. doi: 10.1111/jeb.13307 (PMC6175192; doi:10.1111/jeb.13307)
Supplement: Supplementary file 1 — Figure S1 Results of CAS assay showing per capita iron chelator activities of PA01 WT (cooperator), PA01 cheat, 59.20 WT (cooperator), and 59.20 cheat. Figure S2 Malthusian parameters of strains 59.20 and PA01 with and without the LacZ marker inserted. Table S1 Colony counts, and calculated Malthusian parameters of PA01 cooperator and cheat in competition. Table S2 Colony counts, and calculated Malthusian parameters of PA01 cooperator and 59.20 cheat in competition. Table S3 Colony counts, and calculated Malthusian parameters of 59.20 cooperator and 59.20 cheat in competition. Table S4 Colony counts, and calculated Malthusian parameters of 59.20 cooperator and PA01 cheat in competition. [file JEB-31-1330-s001.docx]

Supplementary figure 1


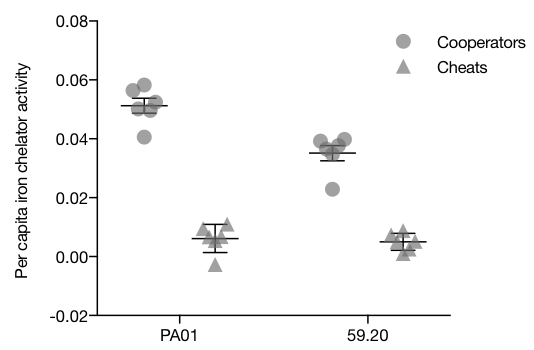


Supplementary figure 1. Results of CAS assay showing per capita iron chelator activities of PA01 WT (cooperator), PA01 cheat, 59.20 WT (cooperator), and 59.20 cheat. Error bars show mean +/- SEM.

Supplementary figure 2


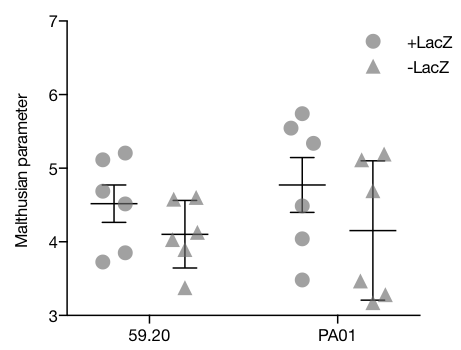


Supplementary figure 2. Malthusian parameters of strains 59.20 and PA01 with and without the *LacZ* marker inserted. Error bars show mean +/- SEM.

Supplementary table 1. Colony counts, and calculated Malthusian parameters of PA01 cooperator and cheat in competition.

| Replicate | Time (hours post inoculation | PA01 cooperator colony count | PA01 cheat colony count | Plated dilution | Cfu/mL PA01 cooperator | Cfu/mL PA01 cheat | M PA01 cooperator | M PA01 cheat |
| --- | --- | --- | --- | --- | --- | --- | --- | --- |
| 1 | 0 | 173 | 76 | -4 | 34600000 | 15200000 |  |  |
| 2 | 0 | 233 | 83 | -4 | 46600000 | 16600000 |  |  |
| 3 | 0 | 189 | 72 | -4 | 37800000 | 14400000 |  |  |
| 4 | 0 | 166 | 75 | -4 | 33200000 | 15000000 |  |  |
| 5 | 0 | 176 | 62 | -4 | 35200000 | 12400000 |  |  |
| 6 | 0 | 157 | 67 | -4 | 31400000 | 13400000 |  |  |
| 1 | 24 | 8 | 22 | -5 | 16000000 | 44000000 | -0.77126496 | 1.06289421 |
| 2 | 24 | 11 | 28 | -5 | 22000000 | 56000000 | -0.75055809 | 1.215949 |
| 3 | 24 | 9 | 26 | -5 | 18000000 | 52000000 | -0.74193734 | 1.28401551 |
| 4 | 24 | 15 | 19 | -5 | 30000000 | 38000000 | -0.10135249 | 0.92953596 |
| 5 | 24 | 9 | 32 | -5 | 18000000 | 64000000 | -0.67067432 | 1.64118661 |
| 6 | 24 | 15 | 23 | -5 | 30000000 | 46000000 | -0.04561051 | 1.23338669 |
| 1 | 48 | 1 | 54 | -5 | 2000000 | 108000000 | 2.525728644 | 5.50311178 |
| 2 | 48 | 1 | 33 | -5 | 2000000 | 66000000 | 2.207274913 | 4.76947324 |
| 3 | 48 | 1 | 34 | -5 | 2000000 | 68000000 | 2.407945609 | 4.87343417 |
| 4 | 48 | 1 | 42 | -5 | 2000000 | 84000000 | 1.897119985 | 5.39840083 |
| 5 | 48 | 1 | 35 | -5 | 2000000 | 70000000 | 2.407945609 | 4.69478234 |
| 6 | 48 | 1 | 62 | -5 | 2000000 | 124000000 | 1.897119985 | 5.59681036 |
| 1 | 72 | 1 | 71 | -5 | 2000000 | 142000000 | 4.605170186 | 4.87886602 |
| 2 | 72 | 1 | 35 | -5 | 2000000 | 70000000 | 4.605170186 | 4.66401069 |
| 3 | 72 | 1 | 60 | -5 | 2000000 | 120000000 | 4.605170186 | 5.17315422 |
| 4 | 72 | 1 | 77 | -5 | 2000000 | 154000000 | 4.605170186 | 5.21130599 |
| 5 | 72 | 1 | 42 | -5 | 2000000 | 84000000 | 4.605170186 | 4.78749174 |
| 6 | 72 | 1 | 80 | -5 | 2000000 | 160000000 | 4.605170186 | 4.86006244 |

Supplementary table 2. Colony counts, and calculated Malthusian parameters of PA01 cooperator and 59.20 cheat in competition.

| Replicate | Time (hours post inoculation | PA01 cooperator colony count | 59.20 cheat colony count | dilution | Cfu/mL PA01 cooperator | Cfu/mL 59.20 cheat | M PA01 cooperator | M 59.20 cheat |
| --- | --- | --- | --- | --- | --- | --- | --- | --- |
| 1 | 0 | 88 | 40 | -3 | 1760000 | 800000 |  |  |
| 2 | 0 | 72 | 56 | -3 | 1440000 | 1120000 |  |  |
| 3 | 0 | 96 | 48 | -3 | 1920000 | 960000 |  |  |
| 4 | 0 | 80 | 56 | -3 | 1600000 | 1120000 |  |  |
| 5 | 0 | 52 | 44 | -3 | 1040000 | 880000 |  |  |
| 6 | 0 | 72 | 72 | -3 | 1440000 | 1440000 |  |  |
| 1 | 24 | 68 | 28 | -5 | 136000000 | 56000000 | 4.347341077 | 4.24849524 |
| 2 | 24 | 84 | 24 | -5 | 168000000 | 48000000 | 4.759320866 | 3.75787233 |
| 3 | 24 | 80 | 16 | -5 | 160000000 | 32000000 | 4.422848629 | 3.5065579 |
| 4 | 24 | 56 | 24 | -5 | 112000000 | 48000000 | 4.248495242 | 3.75787233 |
| 5 | 24 | 44 | 24 | -5 | 88000000 | 48000000 | 4.438116101 | 3.99903438 |
| 6 | 24 | 64 | 32 | -5 | 128000000 | 64000000 | 4.48738715 | 3.79423997 |
| 1 | 48 | 48 | 1 | -4 | 9600000 | 200000 | 1.954278399 | -1.0296194 |
| 2 | 48 | 25 | 1 | -4 | 5000000 | 200000 | 1.090644119 | -0.8754687 |
| 3 | 48 | 25 | 1 | -4 | 5000000 | 200000 | 1.139434283 | -0.4700036 |
| 4 | 48 | 36 | 2 | -4 | 7200000 | 400000 | 1.860752341 | -0.1823216 |
| 5 | 48 | 27 | 3 | -4 | 5400000 | 600000 | 1.814232325 | 0.22314355 |
| 6 | 48 | 15 | 1 | -4 | 3000000 | 200000 | 0.851752211 | -1.1631508 |
| 1 | 72 | 44 | 1 | -3 | 880000 | 20000 | 2.215573716 | 2.30258509 |
| 2 | 72 | 72 | 1 | -3 | 1440000 | 20000 | 3.360375387 | 2.30258509 |
| 3 | 72 | 116 | 4 | -3 | 2320000 | 80000 | 3.837299459 | 3.68887945 |
| 4 | 72 | 88 | 1 | -3 | 1760000 | 20000 | 3.196402969 | 1.60943791 |
| 5 | 72 | 72 | 1 | -3 | 1440000 | 20000 | 3.283414346 | 1.2039728 |
| 6 | 72 | 64 | 1 | -3 | 1280000 | 20000 | 3.753417975 | 2.30258509 |
| 1 | 96 | 36 | 2 | -3 | 720000 | 40000 | 4.404499491 | 5.29831737 |
| 2 | 96 | 32 | 6 | -3 | 640000 | 120000 | 3.79423997 | 6.39692966 |
| 3 | 96 | 88 | 8 | -3 | 1760000 | 160000 | 4.328916809 | 5.29831737 |
| 4 | 96 | 14 | 1 | -3 | 280000 | 20000 | 2.766890701 | 4.60517019 |
| 5 | 96 | 11 | 1 | -3 | 220000 | 20000 | 2.72639934 | 4.60517019 |
| 6 | 96 | 20 | 1 | -3 | 400000 | 20000 | 3.442019376 | 4.60517019 |
| 1 | 120 | 66 | 6 | -3 | 1320000 | 120000 | 5.21130599 | 5.70378247 |
| 2 | 120 | 80 | 1 | -3 | 1600000 | 20000 | 5.521460918 | 2.81341072 |
| 3 | 120 | 76 | 8 | -3 | 1520000 | 160000 | 4.458566712 | 4.60517019 |
| 4 | 120 | 27 | 1 | -3 | 540000 | 20000 | 5.261949722 | 4.60517019 |
| 5 | 120 | 32 | 4 | -3 | 640000 | 80000 | 5.673010816 | 5.99146455 |
| 6 | 120 | 44 | 4 | -3 | 880000 | 80000 | 5.393627546 | 5.99146455 |
| 1 | 144 | 22 | 1 | -4 | 4400000 | 200000 | 5.80914299 | 5.11599581 |
| 2 | 144 | 72 | 1 | -4 | 14400000 | 200000 | 6.802394763 | 6.90775528 |
| 3 | 144 | 184 | 1 | -4 | 36800000 | 200000 | 7.791957696 | 4.82831374 |
| 4 | 144 | 108 | 1 | -4 | 21600000 | 200000 | 8.29404964 | 6.90775528 |
| 5 | 144 | 96 | 8 | -4 | 19200000 | 1600000 | 8.006367568 | 7.60090246 |
| 6 | 144 | 136 | 1 | -4 | 27200000 | 200000 | 8.036220531 | 5.52146092 |

Supplementary table 3. Colony counts, and calculated Malthusian parameters of 59.20 cooperator and 59.20 cheat in competition.

| Replicate | Time (hours post inoculation | 59.20 cooperator colony count | 59.20 cheat colony count | dilution | Cfu/mL 59.20 cooperator | Cfu/mL 59.20 cheat | M 59.20 cooperator | M 59.20 cheat |
| --- | --- | --- | --- | --- | --- | --- | --- | --- |
| 1 | 0 | 22 | 23 | -4 | 4400000 | 4600000 |  |  |
| 2 | 0 | 10 | 9 | -4 | 2000000 | 1800000 |  |  |
| 3 | 0 | 20 | 13 | -4 | 4000000 | 2600000 |  |  |
| 4 | 0 | 7 | 17 | -4 | 1400000 | 3400000 |  |  |
| 5 | 0 | 11 | 10 | -4 | 2200000 | 2000000 |  |  |
| 6 | 0 | 44 | 34 | -4 | 8800000 | 6800000 |  |  |
| 1 | 24 | 4 | 3 | -5 | 8000000 | 6000000 | 0.597837001 | 0.26570317 |
| 2 | 24 | 7 | 5 | -5 | 14000000 | 10000000 | 1.945910149 | 1.71479843 |
| 3 | 24 | 7 | 4 | -5 | 14000000 | 8000000 | 1.252762968 | 1.1239301 |
| 4 | 24 | 1 | 6 | -5 | 2000000 | 12000000 | 0.356674944 | 1.26113122 |
| 5 | 24 | 8 | 9 | -5 | 16000000 | 18000000 | 1.984131362 | 2.19722458 |
| 6 | 24 | 8 | 6 | -5 | 16000000 | 12000000 | 0.597837001 | 0.56798404 |
| 1 | 48 | 6 | 16 | -5 | 12000000 | 32000000 | 5.010635294 | 6.27914662 |
| 2 | 48 | 6 | 29 | -5 | 12000000 | 58000000 | 4.451019506 | 6.3630281 |
| 3 | 48 | 10 | 13 | -5 | 20000000 | 26000000 | 4.96184513 | 5.78382518 |
| 4 | 48 | 1 | 7 | -5 | 2000000 | 14000000 | 4.605170186 | 4.75932087 |
| 5 | 48 | 4 | 9 | -5 | 8000000 | 18000000 | 3.912023005 | 4.60517019 |
| 6 | 48 | 3 | 6 | -5 | 6000000 | 12000000 | 3.624340933 | 4.60517019 |
| 1 | 72 | 1 | 1 | -5 | 2000000 | 2000000 | 2.813410717 | 1.83258146 |
| 2 | 72 | 1 | 5 | -5 | 2000000 | 10000000 | 2.813410717 | 2.84731227 |
| 3 | 72 | 1 | 2 | -5 | 2000000 | 4000000 | 2.302585093 | 2.73336801 |
| 4 | 72 | 3 | 15 | -5 | 6000000 | 30000000 | 5.703782475 | 5.36731024 |
| 5 | 72 | 1 | 3 | -5 | 2000000 | 6000000 | 3.218875825 | 3.5065579 |
| 6 | 72 | 1 | 4 | -5 | 2000000 | 8000000 | 3.506557897 | 4.19970508 |
| 1 | 96 | 2 | 31 | -5 | 4000000 | 62000000 | 5.298317367 | 8.03915739 |
| 2 | 96 | 1 | 9 | -5 | 2000000 | 18000000 | 4.605170186 | 5.19295685 |
| 3 | 96 | 2 | 20 | -5 | 4000000 | 40000000 | 5.298317367 | 6.90775528 |
| 4 | 96 | 3 | 19 | -5 | 6000000 | 38000000 | 4.605170186 | 4.84155896 |
| 5 | 96 | 1 | 15 | -5 | 2000000 | 30000000 | 4.605170186 | 6.2146081 |
| 6 | 96 | 5 | 17 | -5 | 10000000 | 34000000 | 6.214608098 | 6.05208917 |
| 1 | 120 | 3 | 90 | -5 | 6000000 | 180000000 | 5.010635294 | 5.67099265 |
| 2 | 120 | 2 | 84 | -5 | 4000000 | 168000000 | 5.298317367 | 6.83876241 |
| 3 | 120 | 1 | 63 | -5 | 2000000 | 126000000 | 3.912023005 | 5.75257264 |
| 4 | 120 | 9 | 119 | -5 | 18000000 | 238000000 | 5.703782475 | 6.4398547 |
| 5 | 120 | 8 | 190 | -5 | 16000000 | 380000000 | 6.684611728 | 7.14414406 |
| 6 | 120 | 2 | 76 | -5 | 4000000 | 152000000 | 3.688879454 | 6.10269018 |
| 1 | 144 | 1 | 19 | -5 | 2000000 | 38000000 | 3.506557897 | 3.04979949 |
| 2 | 144 | 1 | 7 | -5 | 2000000 | 14000000 | 3.912023005 | 2.12026354 |
| 3 | 144 | 1 | 10 | -5 | 2000000 | 20000000 | 4.605170186 | 2.76462055 |
| 4 | 144 | 1 | 13 | -5 | 2000000 | 26000000 | 2.407945609 | 2.39099605 |
| 5 | 144 | 1 | 6 | -5 | 2000000 | 12000000 | 2.525728644 | 1.14990558 |
| 6 | 144 | 1 | 13 | -5 | 2000000 | 26000000 | 3.912023005 | 2.8393862 |

Supplementary table 4. Colony counts, and calculated Malthusian parameters of 59.20 cooperator and PA01 cheat in competition.

| Replicate | Time (hours post inoculation | 59.20 cooperator colony count | PA01 cheat colony count | dilution | Cfu/mL 59.20 cooperator | Cfu/mL PA01 cheat | M 59.20 cooperator | M PA01 cheat |
| --- | --- | --- | --- | --- | --- | --- | --- | --- |
| 1 | 0 | 64 | 40 | -4 | 12800000 | 8000000 |  |  |
| 2 | 0 | 60 | 64 | -4 | 12000000 | 12800000 |  |  |
| 3 | 0 | 56 | 92 | -4 | 11200000 | 18400000 |  |  |
| 4 | 0 | 80 | 80 | -4 | 16000000 | 16000000 |  |  |
| 5 | 0 | 36 | 28 | -4 | 7200000 | 5600000 |  |  |
| 6 | 0 | 60 | 32 | -4 | 12000000 | 6400000 |  |  |
| 1 | 24 | 35 | 3 | -5 | 70000000 | 6000000 | 1.699050071 | -0.2876821 |
| 2 | 24 | 8 | 8 | -5 | 16000000 | 16000000 | 0.287682072 | 0.22314355 |
| 3 | 24 | 42 | 14 | -5 | 84000000 | 28000000 | 2.014903021 | 0.41985385 |
| 4 | 24 | 56 | 8 | -5 | 112000000 | 16000000 | 1.945910149 | 0 |
| 5 | 24 | 14 | 13 | -5 | 28000000 | 26000000 | 1.358123484 | 1.53532994 |
| 6 | 24 | 48 | 8 | -5 | 96000000 | 16000000 | 2.079441542 | 0.91629073 |
| 1 | 48 | 10 | 2 | -5 | 20000000 | 4000000 | 3.352407217 | 4.19970508 |
| 2 | 48 | 10 | 1 | -5 | 20000000 | 2000000 | 4.828313737 | 2.52572864 |
| 3 | 48 | 8 | 2 | -5 | 16000000 | 4000000 | 2.946942109 | 2.65926004 |
| 4 | 48 | 12 | 2 | -5 | 24000000 | 4000000 | 3.064725145 | 3.21887582 |
| 5 | 48 | 3 | 2 | -5 | 6000000 | 4000000 | 3.064725145 | 2.73336801 |
| 6 | 48 | 15 | 2 | -5 | 30000000 | 4000000 | 3.442019376 | 3.21887582 |
| 1 | 72 | 21 | 1 | -5 | 42000000 | 2000000 | 5.347107531 | 3.91202301 |
| 2 | 72 | 21 | 4 | -5 | 42000000 | 8000000 | 5.347107531 | 5.99146455 |
| 3 | 72 | 12 | 1 | -5 | 24000000 | 2000000 | 5.010635294 | 3.91202301 |
| 4 | 72 | 17 | 1 | -5 | 34000000 | 2000000 | 4.95347688 | 3.91202301 |
| 5 | 72 | 9 | 1 | -5 | 18000000 | 2000000 | 5.703782475 | 3.91202301 |
| 6 | 72 | 10 | 1 | -5 | 20000000 | 2000000 | 4.199705078 | 3.91202301 |
| 1 | 96 | 11 | 1 | -5 | 22000000 | 2000000 | 3.958543021 | 4.60517019 |
| 2 | 96 | 14 | 1 | -5 | 28000000 | 2000000 | 4.199705078 | 3.21887582 |
| 3 | 96 | 8 | 1 | -5 | 16000000 | 2000000 | 4.199705078 | 4.60517019 |
| 4 | 96 | 8 | 1 | -5 | 16000000 | 2000000 | 3.851398384 | 4.60517019 |
| 5 | 96 | 4 | 1 | -5 | 8000000 | 2000000 | 3.79423997 | 4.60517019 |
| 6 | 96 | 22 | 1 | -5 | 44000000 | 2000000 | 5.393627546 | 4.60517019 |
| 1 | 120 | 21 | 1 | -4 | 4200000 | 200000 | 2.949212258 | 2.30258509 |
| 2 | 120 | 11 | 1 | -4 | 2200000 | 200000 | 2.061423036 | 2.30258509 |
| 3 | 120 | 14 | 1 | -4 | 2800000 | 200000 | 2.862200881 | 2.30258509 |
| 4 | 120 | 18 | 1 | -4 | 3600000 | 200000 | 3.113515309 | 2.30258509 |
| 5 | 120 | 26 | 1 | -4 | 5200000 | 200000 | 4.17438727 | 2.30258509 |
| 6 | 120 | 10 | 1 | -4 | 2000000 | 200000 | 1.514127733 | 2.30258509 |
